# Sleep pattern and disorders among pregnant women in Ibadan, Southwest Nigeria

**DOI:** 10.1186/s12905-024-03086-z

**Published:** 2024-04-20

**Authors:** Blessing O Ojelere, Ikeola A. Adeoye

**Affiliations:** 1 Department of Epidemiology and Medical Statistics, Faculty of Public Health, College of Medicine, University of Ibadan, Ibadan, Nigeria; 2 Consortium of Advanced Research for Africa (CARTA), Nairobi, Kenya

**Keywords:** Sleep quality, Insomnia, Restless leg syndrome, Pregnancy, Nigeria

## Abstract

**Background:**

Sleep is essential for pregnant women’s and the offspring’s health and wellbeing. Poor sleep and disorders have been linked with adverse fetal outcomes and delivery conditions. However, pregnant women often experience several forms of sleep disruption, which has been scarcely reported in low and middle-income countries (LMIC), including Nigeria where the influence of lifestyle factors has also been lacking. We investigated sleep patterns and disorders and the associated factors among pregnant women in Southwest, Nigeria.

**Method:**

A cross-sectional study was conducted among five hundred (500) pregnant women attending Adeoyo Maternity Teaching Hospital. A semi-structured questionnaire was used to examine different domains of sleep and associated disorders, namely sleep quality (Pittsburgh Sleep Quality Index (> 5 and ≤ 5)), insomnia (Insomnia Severity Index (> 8 and ≤ 8)), restless leg syndrome (Restless Leg Syndrome Rating Scale (> 10 and ≤ 10). Significant covariates such as physical activity, minimum dietary diversity, smoking and alcohol intake were also assessed. We conducted bivariate and multivariate analysis at *p* < 0.05 significance level.

**Results:**

The mean age of participants was 30.4 ± 4.8 years. The pattern of sleep disorder in pregnant were poor sleep quality (50%), restless leg syndrome (58.2%) and insomnia (33.4%). Being currently married (AOR = 6.13; 95% CI: (1.65–22.23)), increasing gestational age: second trimester (AOR = 8.25;95% CI: (1.78–38.17)) to third trimester (AOR = 10.98; 95% CI: (2.44–49.48)) increased the odds of poor sleep quality. Factors associated with restless leg syndrome were marital status [AOR = 3.60; 95% CI; (1.25–10.35)], religion, rigorous physical activities [AOR = 1.52; 95% CI: (1.05–2.21)] and alcohol consumption [AOR = 3.51; 95% CI: (1.00–12.27)]. Factors associated with insomnia were maternal age [AOR = 1.83; 95% CI: (1.11–3.01)], income [AOR = 2.99 (1.26–7.16)] and rigorous physical activity [AOR = 2.55 (1.61–4.02)].

**Conclusion:**

Poor sleep quality, restless leg syndrome and insomnia were typical among pregnant women in Ibadan, Southwest Nigeria. Thus, awareness and education on the importance of sleep and its risk and protective factors, such as alcohol consumption, smoking, rigorous activity and spousal and family support, should be increased to reduce poor sleep quality and sleep disorders (restless leg syndrome and insomnia) during the pregnancy period.

## Background

Sleep plays a vital role in the wellbeing of pregnant mothers and their babies [1]. Sleep is an innate repeated state of the body and mind characterised by an altered state of consciousness, reduced muscle activity and reduced interactions with the environment [2]. Sleep helps restore the body, cognitive functioning [3] and dreaming [4]. Sleep aids in the regulation of blood sugar and prevention of fatigue and daytime sleepiness; sleeplessness or inadequate sleep can lead to painful labour, low birth weight, preterm delivery, gestational diabetes mellitus, and preeclampsia [5, 6]. However, pregnancy involves some physical, physiological and hormonal adjustment that interferes with sleep [7]. Pregnancy complaints such as frequent urination, nausea, back pain, discomfort from fetal movement, and difficulty in assuming the usual sleep position have a significant effect on sleep patterns [8]. Also, the physiological changes in pregnancy may lead to several signs and symptoms, which can affect their sleep pattern. Sleep deprivation or disturbance can be characterised as sleep onset, insomnia, frequent awakening frequently and waking too early [9].

Sleep disorders are common during pregnancy but are a neglected maternal health issue in Nigeria. Pregnant women are reported to have a higher risk for sleep disorders, with a prevalence of 46–79% and sleep quality declining towards the third trimester [10–12]. Pregnant women also experience poor sleep quality throughout pregnancy [13, 14]. Common sleep disorders during pregnancy include Obstructive Sleep Apnea, Restless Legs Syndrome, Insomnia and gastroesophageal reflux disorder [6, 15]. Some studies also examine some sleep disorders individually in respondents, and the result showed a 36% prevalence of restless leg syndrome and a 32.5% prevalence of insomnia [16, 17]. Poor sleep quality can be linked to stress during pregnancy, antenatal and postpartum depression [18]. A study showed that race, gestational age, and income are associated with poor sleep quality [14]. Other studies also found maternal age, poor economic status, and high-stress levels to increase the risk of sleep problems in pregnant women [9]. Lifestyle issues concerning sleep patterns and disorders have not been reported in most studies, hence this research.

The burden of maternal mortality and morbidity in Nigeria is high, for which several cost-effective interventions have been implemented. However, sleep quality and disorders have been sparsely investigated and considered in Nigeria’s maternal health care. Few studies have reported the prevalence of sleep disorders (insomnia, obstructive sleep apnea, RLS, mild sleepiness, excessive daytime sleepiness and specific awakenings) ranging between 35.5 – 44.1% [19, 20]. With the prevalence of insomnia varying from 32.5% - to 34.6% [18, 20]. A high maternal age and having more children alive were significantly associated with insomnia, especially in the third-trimester, while sleep disorders have been associated with nulliparity, previous adverse obstetric events and increased BMI [17]. This study contributes to the sleep and maternal health literature by assessing sleep patterns and disorders (particularly sleep quality, insomnia and restless leg syndrome) and the associated factors, including lifestyle, behavioural and socio-demographic factors among pregnant women in Ibadan, Nigeria.

## Methods

### Study setting, study population, sample size and selection

A descriptive cross-sectional study was conducted at the Adeoyo Maternity Teaching Hospital (AMTH), Ibadan, Southwest Nigeria, between 11th October and 2nd November 2021. AMTH is a state-owned hospital in Oyo State, Nigeria with comprehensive obstetric care facilities. We recruited five hundred (500) pregnant antenatal care attendees of the hospital. The sample size was determined using the formula for a single proportion and population greater than 10,000 (*n* = Z² p(1-p)/d^2^); n (the minimum sample size), z (standard normal distribution at 95% confidence interval, 1.96), d (maximum value of probability, 0.05) and p (prevalence, 30.8%) non-response rate of 10% resulting into a total of 361 All eligible persons were sampled to obtain the required sample size. Inclusion criteria: those attending ANC between the ages 18 and 49 years, who consented to the study, and exclusion criteria: severely ill and with cognitive dysfunction.

A semi-structured questionnaire was used to examine different domains of sleep: Pittsburgh Sleep Quality Index (sleep quality) (> 5 and ≤ 5), Insomnia Severity Index to ascertain the presence of insomnia and severity, and Restless Leg Syndrome Rating Scale to assess restless leg syndrome. Significant covariates were evaluated: physical activity (the International Physical Activity Questionnaire), dietary patterns (Minimum Dietary Diversity), lifestyle and behavioural factors like smoking and alcohol intake were also assessed as a section for socio-demographic factors and pregnancy-related characteristics. We obtained Ethical approval for the study from the Oyo State Research Ethical Review Committee, Ministry of Health (approval number AD/13/479/4416). We did an ethical review according to guidelines in the Helsinki Declaration Principles 1975, revised in 2000 [21], which was strictly adhered to in this study, and verbal informed consent was obtained from respondents before recruitment into the study.

### Measures

The sleep quality was assessed using the Pittsburgh Sleep Quality Index (PSQI) with an overall score of 21 (> 5 poor quality and ≤ 5 good quality). This instrument also measures sleep latency (time taken to fall asleep within thirty minutes in bed), subjective sleep quality, sleep duration, habitual sleep efficiency (the ratio of the (total sleep time) in a night compared to the total amount of time spent in bed), sleep disturbance (problem initiating and maintaining sleep), use of sleep medication and daytime dysfunction (feeling very sleepy during the day) over one month. The Pittsburgh Sleep Quality Index was developed by Buysse et al. (1989) with specificity (86.5%) and sensitivity (89.6%) [21] and has been validated in Nigeria by Aloba and colleagues [22] and a Cronbach alpha = 0.810.

#### Restless leg syndrome

The Rating Scale was used to assess restless leg syndrome. A total score of less than or equal to 10 indicates an absence of RLS, while greater than 10 indicates the presence of RLS [31–40 (very severe), 21–30 (severe), 11–20 (moderate) and 1–10 (mild)] [23] and Cronbach alpha = 0.937.

#### Insomnia

The Insomnia Severity Index was used to ascertain the presence and severity of insomnia, and a total score of less than or equal to 8 indicates no insomnia, and greater than 8 indicates the presence of insomnia. Severity ranges as follows: 8–14 = Sub threshold insomnia, 15–21 = Clinical insomnia (moderate severity) and 22–28 = Clinical insomnia (severe) [24] and Cronbach alpha = 0.884.

#### Gestational age

This was calculated from the last menstrual period (LMP) and categorised into three trimesters: weeks 1 to 13 (first trimester), 14 to 27 (second trimester) and 28 to 40 (third trimester).

#### Physical activity

The International Physical Activity Questionnaire (IPAQ) assessed physical activity. It measured the average daily time spent sitting, walking, and engaging in moderate and vigorous physical activities over the last seven days. We categorized these activities into vigorous (6–9 METs), moderate (3–6 METs) and light physical activities (1.6–3 METs). The activities’ duration in minutes was multiplied by their estimated value in METs (Metabolic Equivalent Tasks) and summed to gain an overall intensity of physical activity in a week [25] and Cronbach alpha = 0.355.

#### Inadequate diet

Minimum Dietary Diversity for Women (MDD-W) was used to assess adequate and inadequate diet; respondents were asked to recall the food groups they consumed over the previous 24 h. Women who have consumed at least 5 of the ten are classified as having adequate diet diversity; less than 5 indicates inadequate diet diversity [26] and Cronbach alpha = 0.526.

Pre-pregnancy smoking was measured by asking if the pregnant woman ever smoked before the index pregnancy while pregnancy smoking referred to those currently smoking in the current pregnancy. In contrast, second-hand smoke was considered if the husband or household member currently smoked in the woman’s index pregnany. Alcohol consumption was assessed by pre-pregnancy alcohol use and pregnancy alcohol use refered to those consuming alcohol in the current pregnany [27].

### Statistical analysis

Data entry and cleaning were done using Statistical Package for Social Science (SPSS) for Windows version 22.0 (IBM, New York, USA 2013), while data was analysed using STATA version 13 SE (Stata Corp LLC. College Station, TX, USA 2015). We summarized categorical data with frequency distribution and percentages, and used means and standard deviation to summarise continuous data. The dependent variables were sleep quality, restless leg syndrome and insomnia, and the independent variables were – socio-demographic characteristics, obstetric factors and lifestyle and behavioural factors. We assessed risk factors using bivariate logistic regression and reported the odds ratio and 95% confidence interval. For the multivariate analysis, the significant variable (*p*-value < 0.05) in the bivariate were used which included Sleep quality (religion, marital status, parity and gestational age), restless leg syndrome (religion, marital status, employment, rigorous physical activities and pregnancy alcohol consumption) and insomnia (age, religion, income, employment, rigorous physical activity, pre-pregnancy smoking and secondhand smoking).

## Results

### Socio-demographic, lifestyle and behavioural characteristics

Table 1 describes the pregnant women’s background characteristics, including socio-demographic, lifestyle and behavioural factors. The respondents’ mean age was 30.44 ± 4.77 years. The majority of the respondents were between 21 and 40 years old (98.1%), currently married (96.4%), and employed (93.2%). Also, 57.0% were Christians, 67.2% had tertiary education, 13.9% earned less than N20, 000 and 45.2% were nulliparous. Regarding maternal lifestyle, 89% had adequate dietary diversity, and 81.8% engaged in moderate physical activity. Pre-pregnancy smoking was reportedly 3.2%, while smoking during pregnancy was 1.4% and 2.8% were second-hand smokers. Alcohol consumption was 8.6% before pregnancy, but 4.0% during pregnancy.

### Sleep pattern and disorders of pregnant women

**Fig. 1:**
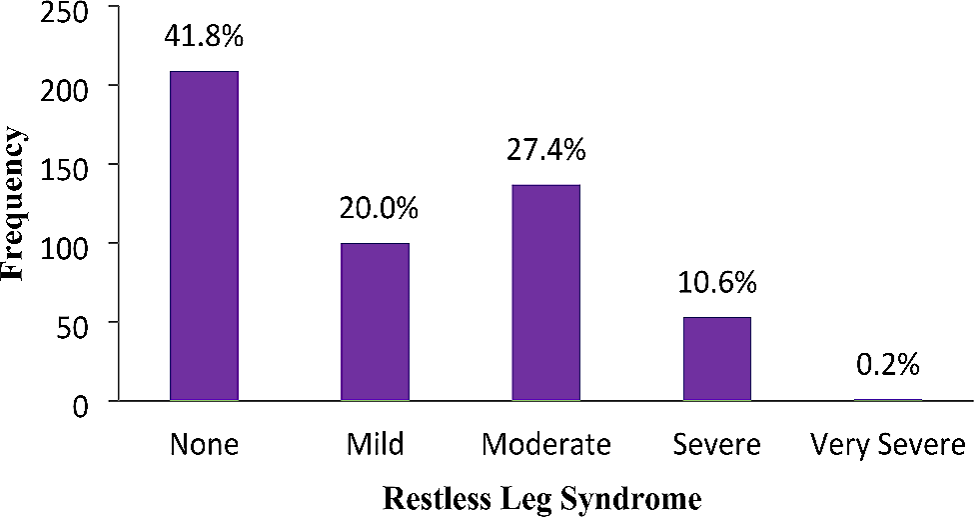
The occurrence of restless leg syndrome among study participants

**Fig. 2:**
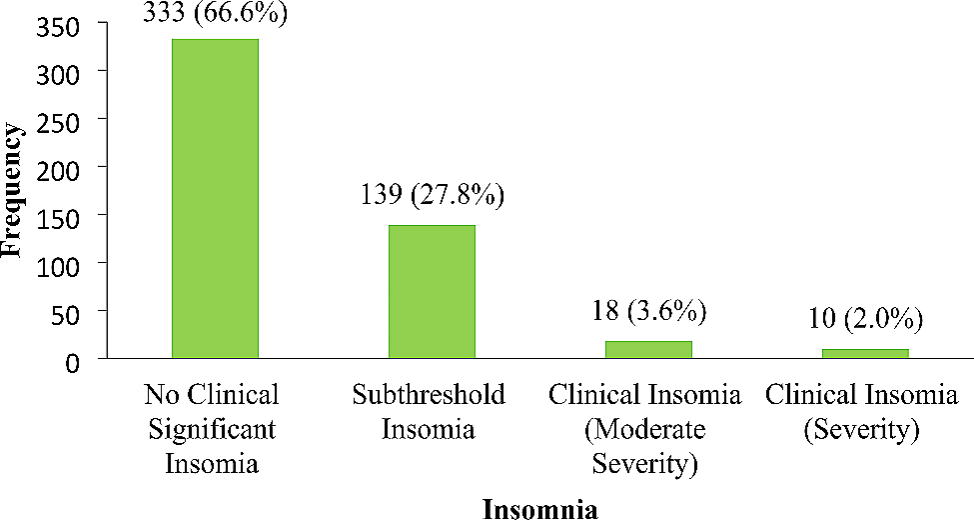
The occurrence of insomnia among study participants

The reported sleep patterns of the women are described in Table 2. About half, 52.4%, reported good quality sleep, 16.2% used sleep medication, and 81.6% reported sleep disturbance. About three-quarters, 78%, had good sleep latency, and 79.6% had good sleep efficiency. Less than half, 45.8% and 23.8%, had daytime dysfunction and short sleep duration, respectively. Overall, 50% had poor sleep quality. Restless Leg Syndrome and Insomnia were reported among pregnant women, 58.2% and 33.4%, respectively (Figs. 1 and 2).

### Factors associated with sleep quality

The factors associated with poor sleep quality are portrayed in Table 3. In the unadjusted logistic model, marital status, religion, parity, and gestational age were significantly associated with poor sleep quality. Notably, being currently married was associated with poor sleep quality [crude Odd Ratio (cOR) = 5.26; 95% confidence interval CI: (1.50 − 18.39); *p*-value: 0.009]. Second and third trimester gestational age increased the odds of poor sleep [cOR = 6.85; 95% CI: 1.49–31.39); *p*-value: 0.013, cOR = 8.15; 95% CI: (1.83–36.18); *P*-value: 0.006]. Conversely, lower odds were associated with being a Muslim [cOR = 0.69; 95% CI: (0.48–0.98); *p*-value: 0.038] and multiparous [cOR = 0.65; 95% CI: (0.42–0.99); *p*-value: 0.048].

In the adjusted model, the odds of poor sleep quality increased with gestational age, second trimester [AOR = 8.25; 95% CI: (1.78–38.17); *p*-value: 0.007] third trimester [AOR = 10.98; 95% CI: (2.44–49.48); *p*-value: 0.003] and being currently married [AOR = 6.13; 95% CI: (1.65–22.23); *p*-value: 0.006] but being a Muslim lowered odds of poor sleep quality by 39% [AOR = 0.61; 95% CI: (0.41–0.89); *p*-value: 0.010] after controlling for other variables.

### Factors associated with restless leg syndromes

The factors associated with restless leg syndrome are shown in Table 4. The unadjusted logistic model indicated that the significant risk factors for restless leg syndrome were religion, marital status, employment status, and rigorous and alcohol intake. Significantly, the risks of RLS were decreased by being a Muslim [cOR = 0.60; 95% CI: (0.42–0.86); *p*-value = 0.006], being employed [cOR = 0.41; 95% CI: (0.18–0.92); *p*-value = 0.030]. In contrast, drinking alcohol when pregnant [cOR = 4.26; 95% CI: 1.23–14.73); *p*-value: 0.022], engaging in rigorous physical activity [cOR = 1.54; 95% CI: 1.07–2.22); *p*-value: 0.019], and being currently married [crude Odd’s Ratio (cOR) = 3.79; 95% CI: 1.33–10.81); *p*-value: 0.013] raised the likelihood of RLS. In the adjusted model, alcohol intake [AOR = 3.51; 95% CI: (1.00–12.27); *p*-value: 0.049], marital status [AOR = 3.60; 95% CI: (1.25–10.35); *p*-value: 0.017] and rigorous physical activities [AOR = 1.52; 95% CI: (1.05–2.21); *p*-value: 0.026] enhanced the odds of RLS.

### Factors associated with insomnia

The variables associated with insomnia are displayed in Table 5. An unadjusted logistic model revealed that associated risk factors for insomnia were age, religion, employment status, income, rigorous physical activity, pre–pregnancy smoking, and second-hand smoking were the significant factors associated with insomnia. In particular, the odds of insomnia decreased by having a religious affiliation, being religious [unadjusted odd’s ratio (cOR) = 0.64; 95% confidence interval CI: (0.44–0.94); *p*-value: 0.024], being employed [cOR = 0.47; 95% CI: (0.24–0.96); *p*-value: 0.037], Conversely, age (older than 35 years) [cOR = 1.97; 95% CI: (1.2–3.14); *p*-value = 0.005], earning 20,000 and above [cOR = 3.44; 95% CI: (1.70–6.96); *p*-value = 0.004], engaging in rigorous physical activity [cOR = 1,85; 95% CI: (1.25–2.75); *p*-value = 0.002], smoking before pregnancy [cOR = 3.47; 95% CI: (1.24–9.72); *p*-value = 0.018] and second-hand smoking [cOR = 12.81; 95% CI: (2.83–57.95); *p*-value = 0.001] increased the odds of insomnia. In the adjusted model, variables associated with insomnia were maternal age, income, second-hand smoking, and rigorous physical activities remained significant. All the significant factors increase the risk of insomnia. Age [AOR = 1.83; 95% CI: (1.11–3.01); *p*-value: 0.018], second hand smoking [AOR = 17.89; 95% CI: (3.54–90.41); *p*-value: <0.001], rigorous physical activities [AOR = 2.55; 95% CI: (1.61–4.02); *p*-value: 0.001] and income of #20,000 -#99,999 [AOR = 3.61; 95% CI: (1.73–7.53); *p*-value: 0.001] income of #100,000 and above [AOR = 2.99; 95% CI: (1.26–7.16); *p*-value: 0.013]

**Table 1:** Characteristics of study participants (*n* = 500)

Socio-demographic	Frequency (N)	Percentage (%)
**Age**		
≤ 20 years	4	0.8
21–30 years	260	55.0
31–40 years	204	43.1
> 40 years	5	1.1
**Mean age ± SD**	30.44 ± 4.77 years	
**Religion**		
Christianity	285	57.0
Islam	215	43.0
**Marital Status**		
Not currently married	18	3.6
Currently Married	482	96.4
**Educational Status**		
Primary	11	2.2
Secondary	153	30.6
Tertiary	336	67.2
**Family Monthly Income**		
< #20,000	69	13.9
#20,000 - #39,999	117	23.6
#40,000 - #79,999	173	34.9
#80,000 - #99,999	63	12.7
≥ #100,000	74	14.9
**Wife Occupation**		
Not Employed	34	6.8
Employed	466	93.2
**Parity**		
Nulliparous	228	45.6
Primiparous	142	28.4
Multiparous	130	26.0
**Lifestyle and Behavioral Characteristics**		
**Dietary diversity**		
Adequate	445	89.0
Inadequate	55	11.0
**Walking**		
No	75	15.0
Yes	425	85.0
**Moderate physical activity**		
No	91	18.2
Yes	409	81.8
**Rigorous physical activity**		
No	197	39.4
Yes	303	60.6
**Pre Pregnancy Smoking**		
No	484	96.8
Yes	16	3.2
**Currently Smoking**		
No	493	98.6
Yes	7	1.4
**Second-hand smoke**		
No	486	97.2
Yes	14	2.8
**Pre Pregnancy Alcohol Consumption**		
No	457	91.4
Yes	43	8.6
**Currently Alcohol Intake**		
No	480	96.0
Yes	20	4.0

**Table 2:** Sleep pattern of the respondents

Sleep Pattern	Frequency (n)	Percentage (%)
**Subjective Sleep Quality**		
Very Good	262	52.4
Fairly Good	215	43.0
Fairly Bad	5	1.0
Very Bad	18	3.6
**Sleep Latency**		
< 15 min(very good)	254	50.8
16–36 min(good)	136	27.2
31–60 min (fairly bad)	78	15.6
>160 min(bad)	32	6.4
**Sleep Duration**		
> 7 h	255	51.0
6–7 h	126	25.2
5–6 h	89	17.8
< 5 h	30	6.0
**Sleep Efficiency (%)**		
> 85%	332	66.4
74–84%	66	13.2
65–74%	44	8.8
< 65%	58	11.6
**Sleep Disturbance**		
0 (No disturbance)	92	18.4
1–9 (mild disturbance)	280	56.0
10–18 (moderate disturbance)	113	22.6
19–27 (severe disturbance)	15	3.0
**Use of sleep medication**		
Not during the past month	419	83.8
Less than once a week	29	5.8
Three or more times a week	52	10.4
**Daytime Dysfunction**		
0 (No)	271	54.2
1–2 (Mild)	119	23.8
3–4 (Moderate)	100	20.0
5–6 (Severe)	10	2.0

**Table 3:** Factors associated with poor sleep quality among pregnant women in Ibadan, Nigeria (Crude and adjusted odds ratios and 95% Confidence Intervals)

	Crude OR (95% CI)	*p*-value	Adjusted OR (95% CI)	*p*-value
Characteristics				
**Age**				
Less than 35	1		1	
35 and above	1.249 (0.79–1.98)	0.344		
**Education**				
Primary or less	1		1	
Secondary	1.64 (0.46–5.83)	0.445		
Tertiary or higher	1.84 (0.53–6.39)	0.340		
**Religion**				
Christianity	1		1	
Islam	0.69 (0.48 - 0.98)	**0.038**	0.61 (0.41–0.89)	0.011*
**Marital status**				
Not currently married	1		1	
Currently married	5.26 (1.50–18.39)	**0.009**	6.13 (1.65–22.23)	0.006*
**Income (monthly income in naira)**				
Less than 20,000	1			
20,000–99,999	1.07 (0.64–1.79)	0.790		
100,000 and above	1.28 (0.66–2.48)	0.457		
**Employment**				
Not employed	1			
Employed	0.78 (0.39–1.56)	0.478		
**Parity**				
Nullipara	1		1	
1–3 (Primiparous)	0.85 (0.56–1.29)	0.460	0.75 (0.50–1.10)	0.147
Four and above (Multiparous)	0.65 (0.42–0.99)	**0.048**	0.40 (0.14–1.23)	0.084
**Gravidity**				
Primigravida	1			
Multigravida	1.02 (0.71–1.46)	0.927		
**Walking**				
No	1			
Yes	1.09 (0.67–1.79)	0.707		
**Moderate physical activity**				
No	1			
Yes	0.67 (0.42–1.05)	0.083		
**Rigorous physical activity**				
No	1			
Yes	0.98 (0.69–1.41)	0.927		
**Dietary diversity**				
Inadequate				
Adequate	1.58 (0.89–2.79)	0.118		
**Pre-pregnancy smoking**				
No	1			
Yes	2.26 (0.77–6.59)	0.137		
**Pregnancy smoking**				
No	1			
Yes	1.34 (0.30–6.04)	0.704		
**Pre – pregnancy alcohol consumption**				
No	1			
Yes	1.16 (0.63–2.18)	0.623		
**Pregnancy alcohol consumption**				
No	1			
Yes	0.65 (0.26–1.63)	0.364		
**Gestational age**				
1st Trimester	1		1	
2nd Trimester	6.85 (1.49–31.39)	**0.013**	8.25 (1.78–38.17)	0.007*
3rd Trimester	8.15 (1.83–36.18)	**0.006**	10.98 (2.44–49.48)	0.003*

**Table 4:** Factors associated with restless leg syndrome among pregnant women in Ibadan, Nigeria (Crude and adjusted odds ratios and 95% Confidence Intervals)

	Crude OR (95% CI)	*p*-value	Adjusted OR (95% CI)	*p*-value
Characteristics				
**Age**				
Less than 35	1			
35 and above	1.38 (0.85–2.23)	0.188		
**Education**				
Primary or less	1			
Secondary	1.18 (0.35–4.05)	0.787		
Tertiary or higher	2.00 (0.59–6.69)	0.260		
**Religion**				
Christianity	1		1	
Islam	0.60 (0.42–0.86)	**0.006**	0.63 (0.43–0.91)	0.013*
**Marital status**				
Not currently married			1	
Currently married	3.79 (1.33–10.81)	**0.013**	3.60 (1.25–10.35)	0.017*
**Income (monthly income in naira)**				
Less than 20,000	1			
20,000–99,999	1.00 (0.59–1.69)	0.987		
100,000 and above	1.06 (0.55–2.07)	0.857		
**Employment**				
Not employed	1		1	
Employed	0.41 (0.18–0.92)	**0.030**	0.46 (0.20–1.07)	0.072
**Parity**				
Nullipara	1			
Primiparous	0.75 (0.49–1.14)	0.173		
Multiparous	0.83 (0.54–1.29)	0.406		
**Gravidity**				
Primigravida	1			
Multigravida	0.85 (0.59–1.23)	0.384		
**Walking**				
No	1			
Yes	1.35 (0.82–2.20)	0.239		
**Moderate physical activity**				
No	1			
Yes	0.75 (0.47–1.21)	0.237		
**Rigorous physical activity**				
No	1		1	
Yes	1.54 (1.07–2.22)	**0.019**	1.52 (1.05–2.21)	0.026*
**Dietary diversity**				
Inadequate	1			
Adequate	0.92 (0.52–1.63)	0.774		
**Pre-pregnancy smoking**				
No	1			
Yes	1.60 (0.55–4.68)	0.389		
**Pregnancy smoking**				
No	1			
Yes	0.53 (0.12–2.41)	0.415		
**Pre-pregnancy alcohol consumption**				
No	1			
Yes	0.99 (0.53–1.88)	0.993		
**Pregnancy alcohol consumption**				
No	1		1	
Yes	4.26 (1.23–14.73)	**0.022**	3.51 (1.00–12.27)	0.049*
**Gestational age**				
1st Trimester	1			
2nd Trimester	0.54 (0.19–1.55)	0.249		
3rd Trimester	0.86 (0.31–2.39)	0.775		

**Table 5:** Shows factors associated with insomnia among pregnant women in Ibadan, Nigeria (Crude and adjusted odds ratios and 95% Confidence Intervals)

	Crude OR (95% CI)	*p*-value	Adjusted OR (95% CI)	*p*-value
Characteristics				
**Age**				
Less than 35	1		1	
35 and above	1.97 (1.2–3.14)	**0.005**	1.83 (1.11–3.01)	0.018*
**Education**				
Primary or less	1			
Secondary	1.29 (0.27–6.24)	0.755		
Tertiary or higher	2.88 (0.61–13.52)	0.181		
**Religion**				
Christianity	1		1	
Islam	0.64 (0.44 - 0.94)	**0.024**	0.85 (0.55–1.29)	0.432
**Marital status**				
Not currently married	1			
Currently married	4.16 (0.95–18.33)	0.059		
**Income (monthly income in naira)**				
Less than 20,000	1			
20,000–99,999	3.44 (1.70–6.96)	**0.001**	3.61 (1.73–7.53)	0.001*
100,000 and above	3.39 (1.49–7.69)	**0.004**	2.99 (1.26–7.16)	0.013*
**Employment**				
Not employed	1			
Employed	0.47 (0.24–0.96)	**0.037**	0.49 (0.23–1.09)	0.081
**Parity**				
Nullipara	1			
Primiparous	1.12 (0.73–1.74)	0.602		
Multiparous	0.69 (0.43–1.12)	0.132		
**Gravidity**				
Primigravida	1			
Multigravida	1.02 (0.69–1.49)	0.928		
**Walking**				
No	1			
Yes	1.00 (0.59–1.69)	0.989		
**Moderate physical activity**				
No	1			
Yes	1.40 (0.85–2.32)	0.186		
**Rigorous physical activity**				
No	1		1	
Yes	1.85 (1.25–2.75)	**0.002**	2.55 (1.61–4.02)	0.001*
**Dietary diversity**				
Inadequate	1			
Adequate	1.53 (0.81–2.90)	0.188		
**Pre-pregnancy smoking**				
No	1		1	
Yes	3.47 (1.24–9.72)	**0.018**	1.47 (0.48–4.51)	0.504
**Pregnancy smoking**				
No	1			
Yes	0.79 (0.15–4.14)	0.785		
**Second-hand smoking**				
No	1		1	
Yes	12.81 (2.83–57.95)	**0.001**	17.89 (3.54–90.41)	<0.001*
**Pre-pregnancy alcohol consumption**				
No	1			
Yes	1.49 (0.78–2.81)	0.221		
**Pregnancy alcohol consumption**				
No	1			
Yes	1.35 (0.54–3.36)	0.524		
**Gestational age**				
1st Trimester	1			
2nd Trimester	1.03 (0.36–3.00)	0.953		
3rd Trimester	0.97 (0.35–2.69)	0.954		

## Discussion

Good sleep quality is essential for pregnant women’s and the offspring’s health and wellbeing. Pregnancy, a period of physiological and emotional changes, influences sleep patterns and could trigger sleep disorders. This study used validated instruments to examine the sleep patterns and sleep disorders of pregnant women receiving antenatal care at Adeoyo Maternity Hospital Ibadan, Nigeria. We found that about half of the study population had poor sleep quality, which was more apparent in the second and third trimesters. A plausible mechanism could be the increased fetal movement and hormonal and anatomical changes as pregnancy progresses. The prevalence of poor sleep quality among our study population was higher than reports from Finland (15%) [28], China (15.2%) [14] and Southwest Ethiopia (30.8%) [29]. On the other hand, a much higher prevalence has been reported by other researchers from Korea (96.2%) [30], Turkey (86%) [8], and Northern Ethiopia (68.4%) [31]. The possible reasons for this discrepancy may be the differences in sample size, eligibility criteria, study setting, socio-demographic, socio-cultural differences, socioeconomic status and environmental issues of the study populations. The Korean study reported a high prevalence (96.2%) of poor sleep quality among participants, possibly due to a mixed population of pregnant and postpartum women. Usually, postpartum women experience poorer sleep quality due to sleep interruptions from the care of the newborn infant [30]. Additionally, studies from developed countries reported higher prevalences of poor sleep quality compared with developing countries due to differences in the social and environmental context, which may affect their sleep hygiene.

This study also revealed significant associations between religion, marital status, gestational age, parity and sleep quality. Similarly, studies in Korea and Northern Ethiopia reported age, gestational age, parity, depression and stress as predictors of poor sleep quality [30, 31]. We found that married women had a six-fold increase (AOR = 6.13) in poor sleep quality. This may be associated with higher household responsibilities such as house chores, childcare activities and the work-life balance [32, 33]. Being married provides social, financial, and emotional support, alleviates stress, and improves sleep quality [34]. However, situations where women are in difficult intimate relationships, such as poor spousal support and domestic violence, may also experience poor sleep quality while being happily married, reduces sleep disturbances [32, 35].

Additionally, the odds of poor sleep quality had a graded response with gestational age, accordingly 2nd trimester: (AOR = 8.25), 3rd trimester: (AOR = 10.98). Pires et al. (2010), who examined the sleep patterns across the three trimesters, observed that sleepiness and reduced sleep efficiency were common in the first trimester, while insomnia and unrestful sleep, and a decrease in REM sleep were common in the second and third trimesters [7]. This is explained by physiological changes that occur as pregnancy progresses, such as breast tenderness and a protruding belly from the enlarging uterus, making it difficult for pregnant women to assume the usual sleep position and affecting sleep quality [5]. Also, increased hormonal secretion, mainly, progesterone and estrogen, decreases the duration of REM sleep in the last trimester [7], whilst the high level of oxytocin in the third trimester induces wakefulness [36]. Anxiety towards the health of the baby and delivery may also increase as pregnancy progresses, leading to poor sleep quality.

The prevalence of insomnia in this study was 33.4%, meaning that one in every three pregnant women experienced insomnia, which corroborated other studies in China (35 -39.6%) [37, 38] and Nigeria 32.5-34.6% [17, 20]. Even then, other studies have reported higher rates of insomnia among pregnant women; Nigeria (47.3%) [19], Turkey (51.2%) and Norway (61.5%) [39, 40]. Variations may come from the study population and setting. The Norway study considered pregnant women in the late stages of pregnancy (≥ 32 weeks gestation). Insomnia has been linked to prolonged labour and increased risk of antepartum depression, especially in the third trimester and after childbirth [11]. The factors associated with insomnia in pregnancy among our study participants were maternal age (≥ 35 years), religion, employment status, high family income, rigorous physical activity, pre-pregnancy smoking and second-hand smoking. However, on multivariate analysis only older maternal age, high family income, rigorous physical activity and second hand smoking remained significant after adjusting for confounders.

We found that older women aged (≥ 35 years) had higher odds of insomnia (AOR = 1.83). Other researchers have also reported the association between high maternal age and insomnia in Nigeria, Turkey and Norway [17, 39, 40]. Older women may have greater household responsibilities, including childcare, which may interfere with or disrupt their sleep. Also, older age is associated with a higher likelihood of co-morbidities such as diabetes, hypertension, and back pain, which may also interfere with sleep [41]. In this study, we found that women earning higher incomes had higher odds of insomnia. High income may be associated with higher work demands and work-related stress. Working late into the night, having a high workload, and using gadgets such as smartphones and computers close to bedtime can make it difficult to fall asleep or interfere with the sleep cycle [42]. Stress from higher income-paying jobs and home responsibilities can lead to insomnia, and stress is recognized as a risk factor for insomnia [11].

We found that rigorous physical activities increased the odds of insomnia twofold, also confirmed by Hartescu and Morgan [43]. Generally, regular and proper physical activity benefits sleep health [44] by establishing a solid sleep-wake cycle, relieving stress and symptoms of sleep disorder and making it easier to fall asleep, thereby reducing the need for sleep medications [45]. However, older age, the type and duration of physical activity can lessen the benefits of physical activity on sleep [44]. For instance, thirty minutes of brisk walking (moderate-intensity exercise) could improve sleep, but rigorous activity (running and weight lifting) would likely hinder sleep [45]. Rigorous physical activities could be counter-productive because they can induce body pain, leg cramps and raised temperature, which can lead to sleep deprivation [46]. Exercise causes the release of endorphins, which increases body temperature, heart rate, and level of alertness and delays sleep onset [47, 48]. Hence, women should engage in moderate intensity physical activity as recommended, but no close to bedtime. Second-hand smokers had higher odds of insomnia. Yolton 2010 also found that exposure to second-hand smoke is associated with more prolonged sleep onset, delayed sleep and overall sleep disturbance [49]. Hashmi et al. also found that passive smoking is a risk factor for insomnia [11]. Smoking generally increases the incidence of insomnia [50]. Night-time smoking or cigarette smoking can trigger sleep disorders such as restless leg syndrome and insomnia [51].

Restless leg syndrome is a condition of the nervous system causing an irresistible urge to move the legs while sitting or sleeping [16]. More than half of our study participants (58.2%) experienced restless leg syndrome, which has scarcely been examined among researchers in Nigeria. The prevalence in this study was higher than reports from the United States (36%) [16] United States (32%) [52] Brazil (13.5%) [53] Saudi (30%) [54] China (11.2|%) [55] and lower than that reported in Turkey (62.7%) [56]. A plausible explanation for variations could be differences in assessment method, study population, sample size and ethnicity, which have been implicated in the occurrence of RLS [53].

This study also showed a significant association between religion, marital status, employment alcohol intake and rigorous physical activities, and further analysis made employment an insignificant risk factor for restless leg syndrome. Being married increases the odds of RLS; a plausible explanation may be numerous house chores and child care, as some authors reported increased parity as a risk factor for RLS [16, 54, 57, 58]. Although studies have shown mild to moderate physical activities like walking, stretching, and swimming to improve RLS symptoms because of increased blood flow to the lower limbs and nitric oxide synthase activity, the release of endorphin and dopamine [59, 60] however, our studies shows that rigorous physical activities (RPA) increase the symptoms of RLS as RPA may cause fatigue and worsen RLS which is similar to some others studies [60, 61].

The WHO (2018) stipulates that there is no safe limit for alcohol consumption during pregnancy; hence, pregnant women must abstain from alcohol during pregnancy [62]. Unfortunately, there is an increase in the intake of alcohol among pregnant women in Africa [63, 64]. Drinking of alcohol among women of reproductive age has been projected to increase in Africa because of economic growth, alteration in gender roles and increasing social acceptability of women’s alcohol consumption [64, 65]. In our study, Alcohol consumption increases the odds of restless leg syndrome by threefold. According to the Center for Vein Restoration, alcohol worsens the itching, pins and needle sensation and other uncomfortable symptoms of RLS and may be associated with increased risk for RLS. Alcohol decreases sleep quality because it disrupts sleep, especially REM sleep, which is vital for the restoration of normal body functioning [66].

Importantly, our study contributes significantly to the literature on sleep health among pregnant women in Nigeria using validated and standardised questionnaires like IPAC, PSQI, etc. We also examined the influence of maternal lifestyle factors such as physical activity, dietary patterns, alcohol consumption, and smoking during pregnancy, which can be targeted for public health interventions. Even then, the study has limitations; being a cross-sectional study, it is not free from temporality bias and cannot elucidate causality. Self-reports assess sleep quality and disorders; hence, there may be recall bias and misclassification bias from measurement errors that result from underreporting or overreporting. Pre-pregnancy or underlying ailments such as maternal obesityhypertension, diabetes, preeclampsia, previous mental disorders and asthma could lead to sleep disorders, and some pregnancy-related conditions such as hydramnios and multi-gestation could cause shortness of breath and could disrupt sleep. Hence, future research should examine the influence of underlying medical co-morbidities and pregnancy complications on sleep patterns and disorders among pregnant women.

## Conclusion

Pregnant women attending antenatal care in Ibadan, Nigeria, had poor sleep quality, insomnia and restless leg syndrome. The factors that influence sleep patterns and disorders maternal age, marital status, gestational age, physical activity, alcohol intake and tobacco use during pregnancy. Therefore, maternal health care providers should address sleep health and disorders as well as maternal lifestyle issues among pregnant women during antenatal care. Significantly, alcohol consumption, exposure to cigarettes and vigorous physical activity should be discouraged for pregnant women. Maternal care professionals, particularly obstetricians, doctors, nurses, midwives, and community health workers, should provide adequate health information on the importance of sleep during pregnancy and antenatal care. Also, screening and treatment of sleep disorders and promoting good sleep hygiene should be provided in maternal healthcare services.

## Data Availability

The datasets generated and analysed during the current study can be obtained from the corresponding author on reasonable request.
